# Antiphospholipid Antibody Syndrome Presenting with Hemichorea

**DOI:** 10.1155/2012/471543

**Published:** 2012-04-11

**Authors:** Yezenash Ayalew, Fazlihakim Khattak

**Affiliations:** Department of Rheumatology, Sandwell General Hospital, Lyndon, West Bromwich, West Midlands B71 4HJ, UK

## Abstract

A 25-year-old Bangladeshi lady presented to neurology with a three-month history of involuntary movements of her right arm, associated with loss of power. There was progression to the right leg, and she subsequently developed episodes of slurred speech and blurred vision. At the time of presentation, she was 12 weeks pregnant and the symptoms were reported to have started at conception. Past medical history was unremarkable apart from one first trimester miscarriage and there was no significant family history suggestive of a hereditary neurological condition. MRI of the head revealed no abnormalities but serology showed positive antinuclear antibodies (ANAs) at a titre of 1/400. Further investigations revealed strongly positive anticardiolipin antibodies (>120) and positive lupus anticoagulant antibodies. The patient had a second miscarriage at 19 weeks gestation strengthening the possibility that the chorea was related to antiphospholipid antibody syndrome and she was started on a reducing dose of Prednisolone 40 mg daily and aspirin 300 mg daily. Six months later, she had complete resolution of neurological symptoms. There are several reports of chorea as a feature of antiphospholipid syndrome, but no clear consensus on underlying pathophysiology.

## 1. Introduction

We would like to report the case of a 25-year-old Bangladeshi lady who was referred to neurology with a three-month history of frequent, involuntary movements of her right arm, with associated loss of power. There was progression to the right leg and she subsequently developed episodes of slurred speech and blurred vision. At the time of presentation, she was 12 weeks pregnant and the symptoms were reported to have started at conception.

Prior to this, she had no other relevant past medical history apart from a first trimester miscarriage. No significant family history suggestive of a hereditary neurological condition was elicited and a working diagnosis of chorea gravidarum was made. 

MRI of the head revealed no abnormalities; however serology showed positive antinuclear antibodies (ANAs) at a titre of 1/400 and a rheumatology opinion was sought. No other features of connective tissue disease were present and however further investigations revealed negative dsDNA antibodies, negative ENA antibodies, and normal compliment C3 but reduced C4 levels. Of particular significance, were the strongly positive anticardiolipin antibodies (>120) and positive lupus anticoagulant tests.

During the course of investigations, the patient had a second miscarriage at 19-week gestation strengthening the possibility that the chorea was related to antiphospholipid antibody syndrome and she was started on oral Prednisolone 40 mg daily and Aspirin 300 mg daily but developed an urticarial rash, which was thought to be related to the latter as the rash resolved on cessation of this drug. Hydroxychloroquine at a dose of 200 mg daily was substituted and she was followed up two weeks later and observed to have had a significant improvement of choreoform movements. The patient was maintained on high-dose steroid therapy for a further two weeks and subsequently weaned off this. Repeat anticardiolipin antibodies and lupus anticoagulant antibodies were still strongly positive twelve weeks later. Six months following her initial presentation, the patient was noted to have complete resolution of her neurological condition. 

## 2. Discussion

Antiphospholipid antibody syndrome (APS) is characterised by recurrent pregnancy loss and thromboembolism due to a procoagulant state conferred by the presence of antiphospholipid antibodies. Involvement of the central nervous system most often presents as a stroke or transient ischaemic attack; chorea is rare in both primary and secondary APS. Currently, the underlying pathophysiology is poorly understood and is not thought to be exclusively explained by a hypercoagulable state [1–3].

Chorea describes an array of symptoms hallmarked by small amplitude and involuntary jerky movements. Such abnormal movements reflect disordered balance of activity in the complex circuitry within the basal ganglia, which in health is responsible for the planning and execution of complex patterns of muscle movements [3–5].

A myriad of conditions can contribute to dysfunction of the basal ganglia, including hereditary disorders such as Huntington's disease, drugs, such as tricyclic antidepressants, and pregnancy.

There are several reports of chorea in the setting of antiphospholipid syndrome, but no clear consensus has been established on the underlying pathophysiology. The published literature is inconclusive about the possible mechanism of injury. *Cervera *et al., in their review of a mixed group of 50 patients with antiphospholipid syndrome and chorea, found that only 35% of the cohort had CT and/or MRI evidence of cerebral infarcts. However, it is worth noting that the cohort was largely made up of patients with SLE, where a vascular pathogenesis is more likely [5].

A possible nonvascular theory about the pathophysiology underlying chorea and other neurological manifestations of APS is an antigen/antibody complex binding phospholipid in the basal ganglia and conferring direct damage to the neurons or supportive tissue. This is bolstered by the lack of positive radiological findings suggestive of an ischaemic insult in our patient.

Chorea should be considered a possible manifestation of APS in young patients and prompt investigations that may reveal primary or secondary APS. Early diagnosis allows for appropriate treatment to minimise complications.
